# Pregnancy and neonatal outcomes of intracytoplasmic sperm injection versus in vitro fertilization in fresh cycles of women with advanced maternal age and nonmale factor infertility: A cross-sectional study

**DOI:** 10.18502/ijrm.v23i1.18190

**Published:** 2025-03-21

**Authors:** Sajad Zare Garizi, Nazanin Sabagh Nezhad Yazd, Nasim Tabibnejad, Razieh Dehghani-Firouzabadi

**Affiliations:** ^1^Student Research Committee, Shahid Sadoughi University of Medical Sciences, Yazd, Iran.; ^2^Research and Clinical Center for Infertility, Yazd Reproductive Sciences Institute, Shahid Sadoughi University of Medical Sciences, Yazd, Iran.

**Keywords:** Infertility, Intracytoplasmic sperm injection, In vitro fertilization, Advanced maternal age, Pregnancy outcome.

## Abstract

**Background:**

Intracytoplasmic sperm injection (ICSI) was originally developed to facilitate fertilization in situations of severe male infertility. However, it is now frequently used for nonmale factor infertility, such as advanced maternal age or low oocyte count, despite the clinical advantages of this method has not been proven for these situations.

**Objective:**

This study aims to compare pregnancy and neonatal outcomes between ICSI and in vitro fertilization (IVF) cycles in women with advanced maternal age and nonmale factor infertility.

**Materials and Methods:**

This retrospective cross-sectional study included 1090 women with nonmale factor infertility, who underwent fresh embryo transfer cycles of IVF or ICSI at the Yazd Reproductive Sciences Institute, Yazd, Iran between April 2018 and June 2023. Data on demographic characteristics, clinical outcomes, and neonatal outcomes were analyzed from electronic medical records.

**Results:**

Women undergoing IVF demonstrated significantly higher outcomes in fertilization, implantation, and chemical pregnancy rate (p < 0.05). Neonatal outcomes showed significantly higher twin birth weights and lower prematurity rates in the IVF group compared to the ICSI group (p < 0.001 and p = 0.011, respectively).

**Conclusion:**

This study suggests that IVF may yield better maternal outcomes and more favorable neonatal results than ICSI for older women with nonmale factor infertility. These results emphasize the significance of tailored treatment plans and the necessity for continued research to enhance assisted reproductive technologies techniques.

## 1. Introduction

Infertility has been identified by the World Health Organization as one of the most significant public health challenges, affecting millions of individuals of reproductive age worldwide, with an estimated 186 million infertile individuals globally (1). With the increasing prevalence of assisted reproductive technologies (ART), public awareness about infertility and its treatments has grown, leading to an annual increase of up to 25% in the number of women seeking infertility services (2).

Advancing maternal age alone negatively impacts female fertility outcomes, as it is associated with higher rates of aneuploidy and spontaneous miscarriage (3). Moreover, advanced maternal age is a well-established factor contributing to reduced oocyte quality and poor in vitro fertilization (IVF) outcomes. To optimize fertilization rates and obtain high-quality embryos in older patients, intracytoplasmic sperm injection (ICSI) has been proposed as an alternative to IVF. The use of ICSI in older couples with nonmale factor infertility is considered logical, as it prevents total fertilization failure caused by insufficient sperm penetration into oocytes, which may be related to maternal age rather than sperm abnormalities. Additionally, ICSI may enhance the number of embryos available at the end of each cycle, potentially improving cumulative pregnancy rates. On the other hand, current scientific evidence does not always confirm these benefits (4–6).

A retrospective study found no advantage of ICSI over IVF in women over 40 yr with nonmale factor infertility (7). Similarly, a 2020 systematic review reported that although ICSI reduces total fertilization failure compared to IVF, it has no significant effect on overall fertilization rates or live birth rates (8). Another study also concluded that ICSI offers no advantage over IVF in achieving live births for couples without male infertility factors (9).

Despite its widespread use, ICSI has limitations. As an invasive procedure, it bypasses the natural selection of oocytes, raising concerns about potential genetic abnormalities. Studies have linked ICSI to a significant risk of congenital anomalies, and infants conceived through ICSI are more likely to require neonatal intensive care unit (NICU) admission (10, 11).

Given the increasing preference for ICSI over IVF, the conflicting results of studies in this domain, and the importance of identifying the most suitable fertility method for women over 35 yr of age, this study aimed to evaluate the pregnancy outcomes between ICSI and conventional IVF cycles among infertile women who referred to the Yazd Reproductive Sciences Institute, Yazd, Iran between April 2018 and June 2023.

## 2. Materials and Methods

This was a retrospective cross-sectional analytical study carried out using data from 5045 infertile women over 35 yr of age with non-male factor infertility who were candidates for IVF or ICSI at the Yazd Reproductive Sciences Institute, Yazd, Iran, between April 2018 and June 2023. The normality of sperm parameters was assessed based on the World Health Organization criteria (12). Participants with diabetes, hypertension, hypothyroidism, hyperthyroidism, pre-eclampsia, eclampsia, body mass index (BMI) ≥ 30 kg/m², malignant ovarian, endometrial, or cervical tumors, or those who underwent cycles involving donor oocytes, split insemination, and preimplantation genetic diagnosis were excluded. Further, women with incomplete medical records were not considered for the analysis.

All relevant data, including demographic and clinical information such as maternal age, maternal BMI, gravidity, parity, infertility type, infertility duration, infertility cause, fertilization method (IVF/ICSI), type of embryo transfer (fresh/frozen embryo transfer), anti-Mullerian hormone concentration, number of retrieved oocytes (the number of eggs collected during the ART process), number of metaphase II oocytes (mature eggs suitable for fertilization), number of 2 pronuclear (2PN) (the number of fertilized eggs showing 2 pronuclei, indicating successful fertilization), fertilization rate (ratio of 2PN oocytes to metaphase II), implantation rate (ratio of embryos that successfully implant in the uterine lining to the total number of embryos transferred), number and quality of transferred embryos, chemical and clinical pregnancy (chemical pregnancy detected by beta-human chorionic gonadotropin levels 2 wk after embryo transfer, and a clinical pregnancy confirmed by ultrasound 4 wk after embryo transfer), ongoing pregnancy (a pregnancy that is progressing beyond 20 wk), singleton or multiple pregnancy, gestational age, preterm delivery (birth occurring before 37 wk of gestation), live birth (a birth where the infant shows signs of life after delivery), abortion (the loss of pregnancy before 20 wk of gestation), stillbirth (the death of a fetus after 20 wk of gestation), ectopic pregnancy, type of delivery, sex of neonate, birth weight, prematurity (birth before 37 wk of gestation), neonatal anomaly, and NICU admission were extracted from hospital electronic medical records.

### Ethical Considerations

This study was approved by the Ethics Committee of Shahid Sadoughi University of Medical Sciences, Yazd, Iran (Code: IR.SSU.MEDICINE.REC.1401.095). All procedures were followed in accordance with the ethical standards of the institutional and national committees, as well as the Helsinki Declaration of 1964.

### Statistical Analysis

The SPSS software (Statistical Package for the Social Sciences, version 27, Chicago, IL, USA) was used for data analysis. Based on the research objectives, the variables were assessed using mean ± standard deviation, N (%), and median (interquartile range). For the final analysis, participants were categorized into 2 groups based on the type of embryo transfer (fresh or frozen). Data from the 2 groups (IVF and ICSI) were then analyzed and compared. Qualitative variables were assessed using the Chi-square test, and Fisher's exact test was applied when required. Quantitative variables were analyzed using the Student's *t* test and Mann-Whitney U test. Linear regression analysis was performed to assess the association between age and the number of retrieved oocytes. Multivariable logistic regression analysis was conducted to evaluate the association between age and live birth, abortion, stillbirth, and prematurity. A p-value of < 0.05 was considered statistically significant.

## 3. Results

A total of 5045 infertile women aged over 35 yr with nonmale factor infertility who underwent IVF/ICSI cycles were identified and assessed for eligibility in this study. Among these, 489 women were excluded due to missing information, 188 were attributed to underlying medical conditions, 911 for undergoing cycles involving donor oocytes, 854 for split insemination, and 436 for having a BMI over 30. After applying these exclusion criteria, data from 2167 women were included. Of these, 1090 underwent fresh embryo transfer, while 1077 underwent frozen embryo transfer. This study focuses exclusively on outcomes from fresh embryo transfers.

A total of 1090 participants were included in the final analysis, with 97 (8.90%) undergoing IVF and 993 (91.10%) undergoing ICSI. The mean age of women undergoing IVF was 38.00 ± 2.18 yr, which was younger than the mean age of women undergoing ICSI 40.14 ± 3.45 (p < 0.001). Other demographic characteristics and treatment cycle details for the IVF and ICSI groups are listed in table I. For some variables such as gravidity, parity, infertility type, infertility cause, and embryo grade in both groups, data were unavailable for all cases in the reviewed medical records. Therefore, the numbers and percentages presented in the tables are based on the cases with complete data.

The findings revealed that the IVF group demonstrated significantly higher outcomes for the number of 2PNs, embryo grading, fertilization rate, implantation rate, and chemical pregnancy rate compared to the ICSI group (p < 0.05, Table II).

Neonatal outcomes for the IVF and ICSI groups are presented in table III. The results demonstrate that the mean birth weight of twins in the IVF group was significantly higher than that in the ICSI group (p < 0.001). Furthermore, the prematurity rate among twins was significantly lower in the IVF group compared to the ICSI group (p = 0.011).

The results of the linear regression analysis indicated that the coefficient for the variable “age" was -0.121, suggesting that 1-yr increase in age corresponds to a 0.12-unit decrease in the log odds of retrieved oocytes (p = 0.001). The correlation between age and retrieved oocytes was 0.105, indicating a weak positive correlation (Table IV).

In addition, to adjust for confounding factors, we performed a logistic regression analysis to examine the relationship between age and live birth rate, abortion, stillbirth, and prematurity. The logistic regression analysis revealed no significant associations between age and live birth rate, abortion, stillbirth, or prematurity (p > 0.05). Specifically, for live birth rate, each one-unit increase in age was associated with a 0.073 decrease in the log odds, corresponding to a 7% reduction in the odds (OR = 0.930). Similarly, age was linked to an 8% increase in the odds of abortion (OR = 1.080), a 3.6% increase in the odds of stillbirth (OR = 0.964), and a 3.7% increase in the odds of prematurity (OR = 0.963). However, none of these associations reached statistical significance. In contrast, twin pregnancies were significantly associated with prematurity, with mothers of twins having 6.8 times higher odds of prematurity compared to mothers of singletons (OR = 6.806, p = 0.001) (Table V).

**Table 1:** Women's demographic and treatment cycle characteristics in 2 groups

**Variables**		**IVF (n = 97)**	**ICSI (n = 993)**	**P-value**
Maternal age (yr)*		38.00 ± 2.18 (38, 3)	40.14 ± 3.45 (40, 6)	< 0.001
Maternal BMI (kg/m^2^)*		24.26 ± 2.97 (24.65, 4)	25.31 ± 2.78 (25.72, 4)	0.310
Gravidity**				
	**0**	31 (44.93)	326 (69.36)	
Gravidity**				
	**1**	17 (24.64)	108 (22.98)	0.006
	**≥ 2**	21 (30.43)	36 (7.66)	
Parity**				
	**0**	57 (82.61)	458 (88.08)	0.198
	**≥ 1**	12 (17.39)	62 (11.92)	
Infertility type**				
	**Primary**	31 (44.29)	327 (62.52)	0.003
	**Secondary**	39 (55.71)	196 (37.48)	
Infertility duration (yr)*		5.37 ± 3.31 (5, 4.3)	6.26 ± 4.62 (5, 6.5)	0.910
Infertility cause**				
	**PCOS**	23 (46.94)	161 (46.67)	0.011
	**POF**	10 (20.41)	92 (26.67)	
	**Tubal factor**	7 (14.28)	12 (3.48)	
	**Endometriosis**	0 (0)	14 (4.05)	
	**Unexplained**	9 (18.37)	66 (19.13)	
AMH (ng/ml)*		2.80 ± 2.38 (2.10, 2.7)	2.26 ± 2.41 (1.50, 2.1)	0.178
Number of retrieved oocytes*		7.35 ± 2.51 (7, 4)	5.18 ± 3.04 (5, 4)	< 0.001
Number of MII oocytes*		6.57 ± 2.56 (6, 5)	4.11 ± 2.53 (4, 3)	< 0.001
*Data are presented as Mean ± SD (median, interquartile range). Mann-Whitney U test. **Data are presented as n (%). Chi-square test. IVF: In vitro fertilization, ICSI: Intracytoplasmic sperm injection, BMI: Body mass index, PCOS: Polycystic ovary syndrome, POF: Premature ovarian failure, AMH: Anti-Mullerian hormone, MII: Metaphase II				

**Table 2:** Treatment cycle characteristics in 2 groups

**Variables**		**IVF (n = 97)**	**ICSI (n = 993)**	**P-value**
Number of 2PNs*		5.54 ± 2.55 (6, 4)	2.62 ± 1.71 (2, 3)	< 0.001
Fertilization rate**		398/498 (79.92)	2873/5677 (50.61)	< 0.001
Implantation rate**		20/151 (13.24)	102/1654 (6.17)	< 0.001
Transferred embryo*		1.97 ± 0.16 (2, 0)	1.63 ± 0.48 (2, 1)	< 0.001
Embryo grade**				
	**A**	22 (28.95)	168 (17.59)	< 0.001
	**AB**	20 (26.32)	161 (16.86)	
	**AC**	1 (1.31)	14 (1.47)	
	**B**	24 (31.58)	351 (36.75)	
	**BC**	7 (9.21)	134 (14.03)	
	**BD**	0 (0)	2 (0.21)	
	**C**	2 (2.63)	115 (12.04)	
	**CD**	0 (0)	4 (0.42)	
	**D**	0 (0)	6 (0.63)	
Chemical pregnancy**		33 (34.02)	184 (18.53)	< 0.001
Clinical pregnancy**		22/33 (66.67)	124/184 (67.39)	0.935
Ongoing pregnancy**		19/33 (57.58)	103/184 (55.98)	0.865
Live birth**		16/33 (48.48)	93/184 (50.54)	0.828
Abortion**		15/33 (45.45)	86/184 (46.74)	0.892
Stillbirth**		2/33 (6.06)	5/184 (2.72)	0.317
EP**		0/33 (0)	3/184 (1.63)	0.460
Gestational age (yr)*		37.97 ± 1.74 (38, 6)	37.44 ± 1.74 (38, 9)	0.222
Preterm delivery**		2/16 (12.50)	24/93 (25.81)	0.249
Type of delivery**				
	**NVD**	2 (12.50)	13 (13.98)	0.874
	**C/S**	14 (87.50)	80 (86.02)	
Singleton or multiple pregnancy**				
	**Singleton**	12 (75.00)	84 (90.32)	0.081
	**Twins**	4 (25.00)	9 (9.68)	
*Data are presented as Mean ± SD (median, interquartile range). Mann-Whitney U test. **Data are presented as n (%). Chi-square test. IVF: In vitro fertilization, ICSI: Intracytoplasmic sperm injection, 2PN: 2 pronuclear, EP: Ectopic pregnancy, NVD: Normal vaginal delivery, C/S: Cesarean section				

**Table 3:** Neonatal outcomes according to fertilization methods in 2 groups

**Variables**			**IVF (n = 20)**	**ICSI (n = 102)**	**P-value**
Sex of neonate*					
	**Singleton**				
		**Girl**	4 (33.33)	47 (55.95)	
		**Boy**	8 (66.67)	37 (44.05)	0.142
	**Twins**				
		**Girl**	3 (37.50)	9 (50.00)	
		**Boy**	5 (62.50)	9 (50.00)	0.555
Birth weight (gr)**					
	**Singleton**		2890.00 ± 558.83	2971.25 ± 508.23	0.610
	**Twins**		2786.25 ± 216.65	2103.89 ± 404.31	< 0.001
Prematurity*					
	**Singleton**		1/12 (8.33)	17/84 (20.24)	0.323
	**Twins**		2/8 (25.00)	14/18 (77.78)	0.011
NICU admission*					
	**Singleton**		0 (0)	6/84 (7.14)	0.339
	**Twins**		2/8 (25.00)	6/18 (33.33)	0.671
Neonatal anomaly*					
	**Singleton**		0 (0)	1/84 (1.19)	0.704
	**Twins**		1/8 (12.50)	0 (0)	0.126
*Data are presented as n (%). Chi-square test. **Data presented as Mean ± SD. Independent *t* test. IVF: In vitro fertilization, ICSI: Intracytoplasmic sperm injection, NICU: Neonatal intensive care unit					

**Table 4:** Linear regression results adjusting the effect of age on retrieved oocytes

**Variable**	**Coefficient**	**Standard error**	***t *** **test statistic**	**P-value**	**R^2^**
**Retrieved oocytes**	- 0.121	0.035	-3.424	0.001	0.930

**Table 5:** Results of multiple regression analysis of live birth, abortion, stillbirth, and prematurity

**Variables**		**Coefficient**	**Standard error**	**Wald-test statistic**	**P-value**	**Odds ratio**
**Live birth**						
	**Age**	-0.073	0.041	3.185	0.074	0.930
**Abortion**						
	**Age**	0.077	0.041	3.607	0.058	1.080
**Stillbirth**						
	**Age**	-0.037	0.117	0.101	0.751	0.964
**Prematurity**						
	**Age**	-0.038	0.063	0.363	0.547	0.963
	**Singleton/twins**	1.918	0.482	15.850	0.001	6.806

## 4. Discussion

This study focused on creating ICSI to help couples facing severe male infertility issues where IVF was not an option. As expected, our results showed a significantly higher number of ICSI cycles, with a distinct age profile compared to IVF cycles. Over time, this technique has been widely adopted for other infertility causes, even in cases with normal semen parameters (4). However, despite the controversies regarding the advantages and safety of ICSI, this technique is now widely used in various conditions, including low oocyte yield, previous fertilization failure with IVF, and advanced maternal age (13).

While most comparative studies between IVF and ICSI focus on fertilization rates and live birth outcomes (3–5, 7–9, 13), limited attention has been paid to pregnancy and neonatal outcomes, particularly in fresh cycles among older women with nonmale factor infertility. This gap in the literature motivated the present study, which analyzed outcomes in a population of infertile women over 35 yr old with nonmale factor infertility undergoing IVF or ICSI.

Our findings demonstrated that IVF outperformed ICSI in several key metrics, including 2PN embryos, fertilization rate, implantation rate, and chemical pregnancy rate. Additionally, neonatal outcomes favored the IVF group, with significantly higher twin birth weights and lower rates of prematurity among twins. These results challenge the routine application of ICSI for nonmale factor infertility and suggest that IVF may offer advantages in this context.

Literature comparing ICSI and IVF in older women with nonmale factor infertility presents conflicting results. Some studies report higher fertilization rates in ICSI cycles (3, 14, 15), while others indicate comparable or even superior outcomes with IVF (7, 16–18). These discrepancies may be attributed to variations in inclusion criteria, study designs, and patient populations across studies.

In the present study, the implantation rate was significantly higher in the IVF group compared to the ICSI group (13.24% vs. 6.17%). In the study by Kim and colleagues, albeit the implantation rate was higher in the IVF group relative to the ICSI group (25% vs. 15%), this difference was statistically insignificant (19). Another study, when analyzing subgroups, found no significant difference in implantation rates between cycles with a low number of oocytes ( ≤ 4 oocytes) and cycles with older mothers ( ≥ 40 yr) for both IVF and ICSI. Nevertheless, IVF performed better than ICSI in cycles with both a low number of oocytes and older maternal age ( ≤ 4 oocytes and ≥ 40 yr), showing implantation rates of 11.7% vs. 2.6% (p = 0.027) (14).

The selection criteria for ICSI in this study may partially explain the observed differences in outcomes. Women undergoing ICSI were generally older and had a longer duration of infertility, suggesting more severe reproductive challenges. Despite controlling for baseline characteristics, unmeasured confounding factors may have influenced outcomes. This underscores the need for randomized controlled trials to identify specific subgroups that could benefit from ICSI.

In the present study, the distribution analysis of singletons and multiples indicated that ICSI resulted in a higher percentage of singletons compared to IVF, which may be due to the transfer of a higher number of embryos in the IVF group. Although this difference was not significant, it aligns with the findings of Liu and colleagues (4).

Interestingly, neonatal outcomes in this study were consistent with prior research. Birth weight and gestational age were comparable between IVF and ICSI-conceived infants, and no significant differences were observed in congenital anomalies or NICU admission rates (4, 20). A study performed in China on 15,405 ART-conceived children indicated that the risk of congenital malformations in the ICSI group was similar to that in the conventional IVF group (21). While reviewing the existing literature, there are several opinions given the risks of hospitalization of IVF- and ICSI-conceived infants in the NICU. Nouri et al. found that infants conceived via ICSI were admitted to the NICU more often, while other studies have shown differing outcomes (11). In a large cohort study including 2889 ICSI-conceived infants and 2995 IVF-conceived infants, the rate of NICU admission was higher among the ICSI group than the IVF group (22). However, variations in NICU admission rates reported across studies may reflect differences in healthcare systems, subjective criteria for admission, and cultural attitudes toward “precious pregnancies" resulting from ART (4).

One of the strengths of this study was the separation of fresh and frozen embryo transfers, with this paper focusing solely on the results of fresh embryo transfers. Be that as it may, the retrospective design and the lack of randomization limit the ability to draw definitive conclusions. The present study is cross-sectional and is by no means designed to identify cause-and-effect relationships. A well-known limitation of retrospective cross-sectional studies is the influence of confounding variables, which was also a key limitation of our study. To minimize the impact of confounding factors, we used logistic regression to assess the effect of age on key variables influenced by age in the context of fertility. Some participants had missing data and we made sure to complete the data as much as possible, but certain gaps remained beyond our control. Further prospective, adequately powered studies are needed to validate these findings and refine patient selection criteria for ICSI.

## 5. Conclusion

This study suggests that IVF may offer superior maternal outcomes and favorable neonatal results compared to ICSI for older women with nonmale factor infertility. These findings emphasize the need for individualized treatment approaches and highlight the importance of further research to optimize ART strategies.

##  Data Availability

Data supporting the findings of this study are available upon reasonable request from the corresponding author.

##  Author Contributions

N. Tabibnejad and S. Zare Garizi, designed the study and conducted the research. Monitoring, evaluating, and analyzing the data were done by all authors. Also, all authors reviewed the article, approved the final manuscript, and take responsibility for the integrity of the data.

To ensure fairness and accurately reflect contributions, this article designates 2 corresponding authors, who jointly designed the study, conducted the data analysis, and contributed to drafting the manuscript. This dual authorship structure for both corresponding authors is essential to acknowledge the balanced and collaborative efforts that were central to this study's success.

##  Conflict of Interest

The authors declare that there is no conflict of interest.
